# Outcome of the entomological monitoring for Crimean-Congo haemorrhagic fever virus in the western and southern regions of Kazakhstan in 2021–2022

**DOI:** 10.3389/fepid.2024.1310071

**Published:** 2024-08-22

**Authors:** T. Nurmakhanov, N. Tukhanova, Z. Sayakova, V. Sadovskaya, A. Shevtsov, G. Tokmurziyeva, N. Turebekov

**Affiliations:** ^1^ Laboratory of Natural-Focal Viral Infections, M.Aikymbayev's National Scientific Center for Especially Dangerous Infections, Almaty, Kazakhstan; ^2^ Department of Epizootology of Especially Dangerous Infections with the Museum and Insectarium, M.Aikymbayev's National Scientific Center for Especially Dangerous Infections, Almaty, Kazakhstan; ^3^ Department of Biostatistics and GIS, M.Aikymbayev's National Scientific Center for Especially Dangerous Infections, Almaty, Kazakhstan; ^4^ Laboratory of Applied Genetics, National Center for Biotechnology, Astana, Kazakhstan; ^5^ M.Aikymbayev's National Scientific Center for Especially Dangerous Infections, Almaty, Kazakhstan

**Keywords:** ticks, Crimean - Congo haemorrhagic fever virus, S, M and L segments, phylogenetic analysis, natural foci, Kazakhstan

## Abstract

The natural foci of Crimean-Congo haemorrhagic fever (CCHF) in Kazakhstan are geographically located in the southern regions of the country (Kyzylorda, Turkestan and Zhambyl regions), where the infection of ticks with the CCHF virus predominantly reside, tick species composition and the number of vectors are monitored annually. The objective of our research was to investigate the genetic variants of the CCHF virus in the southern endemic regions, as well as to monitor the spread of the CCHF virus in the western regions of the country (Aktobe, Atyrau and Mangystau regions). In total, 974 (216 pools) ticks from the western regions and 3527 (583 pools) ticks from the southern regions collected during 2021–2022 were investigated. The presence of CCHF virus was detected by real-time reverse transcription PCR (qRT- PCR) in 1 pool out of 799 pools (0.12%) with *Hyalomma scupense* ticks captured in the CCHF-endemic Kyzylorda region. In the western regions, CCHF virus was not detected in ticks. The sequencing of incomplete fragments of the S, M and L segments of the CCHF virus in the detected virus was identified as genotype *Asia - I*. Phylogenetic analysis showed that the isolate obtained in this study is grouped with the isolate from a patient with CCHF, which we reported in 2015 (KX129738 Genbank). Our findings highlight the importance of including sequencing in the annual monitoring system for better understanding the evolution of the CCHF virus in the study areas of our country.

## Introduction

Emerging and re-emerging human viral infections are one of the global health challenges (1–3). The gravity of such infections is due to the sudden appearance, rapid spread and often the lack of specific protection and prevention. The main source of new human viruses are zoonotic viruses, which, under the influence of a combination of factors, acquire human-pathogenic properties and epidemic potential (4, 5). A significant proportion of the causative agents of these infections are arboviruses, mainly containing RNA, which are transmitted to humans through blood-sucking arthropod vectors. The Crimean-Congo haemorrhagic fever virus (CCHF) belongs to the genus *Orthonairovirus*, family *Nairoviridae*, order *Bunyavirales* (6). Due to the fact that the majority of modern vaccines are at the stage of development and clinical trials (7), and the strategy for creating effective antiviral drugs is experiencing certain difficulties along the way (8), according to the WHO, this disease is considered to be a potential public health emergency. There are several classes of drugs with antiviral activities against different viruses, including CCHF virus, such as nucleoside analogues (ribavirin, favipiravir, 2′-deoxy-2′-fluorocytidine), ovarian tumor (OTU) domain proteases (phenanthrenequinone compounds), interferons, immunotherapy (convalescent serum, monoclonal antibodies) and a few anti-malarial and anti-psychotic drugs (chloroquine and chlorpromazine) (8). Ribavirin is widely used in Kazakhstan as anti-CCHF viral medicine due to its low cost and accessibility, and is considered the drug of choice in our country.

Crimean-Congo haemorrhagic fever is one of the most significant natural focal viral infections in Kazakhstan. Outbreaks of the disease are annually reported in three endemic regions (Kyzylorda, Turkestan and Zhambyl regions) geographically located in the southern part of the country. The main vectors of CCHF virus in Kazakhstan are ticks of the *Hyalomma* genus (*Hyalomma asiaticum, H.scupense, H.anatolicum*). These tick species are pasture-stall arthropods with a two- or three-host life cycle. The natural habitat of the *Hyalomma* spp. in Kazakhstan are deserts and steppes. These ticks are also successfully adapted to inhabit in populated areas and their environs. These arthropods actively parasitize both domestic cattle and small ruminants, as well as wild animals (kulans, saigas, goitered gazelles, etc.) in Kazakhstan (9).

The committee for veterinary control and supervision of the Ministry of Agriculture and national centers of expertise together with anti-plague stations of the Ministry of Health are responsible for monitoring of situations around CCHF in Kazakhstan. The annual treatment of farm animals with pesticides in CCHF foci does not give the proper level of infection reduction, and the increased economic activity of the local population in CCHF natural foci in recent years has led to an increase in human contacts with arthropod vectors that are not only the vectors but also the reservoirs of the virus. As a result, human cases of CCHF are being reported in areas where the disease has not been seen for many decades. We set the following tasks for the implementation of the national scientific program: to collect ticks in the southern regions endemic for CCHF (Kyzylorda, Turkestan and Zhambyl regions) and non-endemic western regions (Aktobe, Atyrau and Mangystau regions), to determine the species composition of captured ticks, to monitor the infection of ticks with the CCHF virus by PCR, followed by sequencing of isolated virus isolates. The aim of our study was to investigate the ticks, collected from the southern endemic regions and western non-endemic regions for the presence of the genetic variants of the CCHF virus.

## Materials and methods

### Tick collection and species identification

Trapping of ticks was carried out in open areas of the countryside where there is vegetation, using the “flag” method in the southern (Kyzylorda, Turkestan and Zhambyl) and western (Aktobe, Atyrau and Mangystau) regions of Kazakhstan in April, May and June of 2021–2022. As a flag, a cotton fabric measuring 0.6 × 1.0 m was used, the narrow side of which was attached to a stick 1.25–1.50 m long. Every 5 m, the fabric was inspected for the presence of ticks. The collection of ticks from farm animals was carried out mainly in the yards of the local population and in private livestock farms. To do this, the animal was fixed and examined in typical places of tick bites: in the udder, on the inside of the thighs and in the groin. Ticks were removed with tweezers and collected into plastic tubes with a tightly screw lid. Animal housing facilities were also inspected for the presence of ticks, mainly fenced pens where animals are kept after they return from pasture. In the western regions of Kazakhstan (Aktobe, Atyrau and Mangystau regions), ticks were mainly removed from farm animals due to unstable weather in spring, accompanied by a sharp decrease and increase in temperature, and precipitation. The collected ticks were stored in the tubes at −20°C until further analysis. Species identification of ticks was carried out according to manuals (10–12) using a binocular stereoscopic microscope and by morphological features such as the pattern and shape of the scutum, festoons, capitulum, hypostome, palps, eyes, peritreme, legs etc. After that the ticks were grouped into pools of 5–10 ticks of the same species each. Geographic information system technologies were used for mapping the locations where ticks were collected. Electronic maps of Kazakhstan were applied as a topographic basis and included the following base layers: mathematical elements, hydrography and administrative structure. The obtained information on ticks was digitized by creating electronic databases using Excel program, and then adapted to work in the ArcMap program.

### Viral RNA extraction from ticks

Tick pools were homogenized using a “TissueLyser II” (Qiagen, Hilden, Germany) homogenizer in 6 min at 300 Hz with the addition of stainless steel beads and 1 ml of cell growth medium (DMEM, Gibco™, Thermo Fisher Scientific, Waltham, MA, USA) to each sample. RNA was extracted from 140 μl tick homogenates using the commercial kit (QIAamp Viral RNAMini Kit; Qiagen, Hilden, Germany), according to the manufacturer's instruction and stored at −80°C.

### Real-time reverse transcription PCR (qRT-PCR)

All PCR procedures were conducted in a three- room regime. The presence of CCHF viral RNA was determined according to the protocol from B. Atkinson et al. (13) for qRT-PCR using the LightCycler 2.0 instrument (Roche Applied Science, Switzerland). We used SuperScript III (SSIII) Platinum One-step qRT-PCR Kit (Invitrogen) to prepare the reaction mixture. The volume of the master mix (15 μl) included 10 μl of 2× reaction mixture, 1.7 μl of PCR-grade water, 1 μl of each primer (for 18 µM working concentration), 0.5 µl probe (for 25 µM working concentration) and 0.8 µl SuperScrip Taq Mix. After that, 5 μl of RNAwas added to the master mix to obtain a final reaction volume of 20 μl. The applied amplification program: at 50°C for 10 min., at 95°C for 2 min., then 45 cycles of 95°C for 10 s and at 60°C for 40 s (with fluorescence quantitation at the end of each step at 60°C) and the final stage of cooling at 40°C for 30 s.

#### Reverse transcription and sequencing

The extracted RNAsamples were converted into cDNAby reverse transcription using the commercial kit (Reverta L; AmpliSens, Russia). The S, L and M segments fragments were amplified using two-round PCR with CCHF virus-specific primers (Table 1) (14, 15). PCR was conducted in a volume of 30 µl. The reaction mixture contained 0.65-µm concentration of segment-specific primers for each round, 200-µm concentration of each deoxynucleoside triphosphate, 2.5 mm MgCl_2_ 1× PCR buffer, 1× Q-Solution 1.5 U HotstarTaq plus DNA polymerase (Qiagen, Valencia, CA, USA), and 5 µl of cDNA or 5 µl of the product obtained from the first round of PCR. PCR was carried out with a “T100™ Thermal Cycler” (Bio-Rad, USA) by using the following temperature program—at 95°C for 5 min; 35 cycles: at 95°C for 25 s, at 58°C (at 49°C for the second round of PCR) for 1 min; and final polymerization at 72°C for 10 min. PCR products of the first and second stages were subjected to electrophoretic separation in 1.5% agarose. All amplified target fragments were sequenced. PCR products were purified with exonuclease I (Exo I, Thermo Fisher Scientific Baltics UAB) and alkaline phosphatase (Shrimp Alkaline Phosphatase, Thermo Fisher Scientific Baltics UAB) as described earlier (16). Purified PCR products were labeled with fluorescent dyes using BigDye Terminator v3.1 Cycle Sequencing Kit (Applied Biosystems™, USA). Samples were sequenced using an ABI 3730xl DNA Analyzer (Applied Biosystems™, USA). SeqMan II software (Lasergene, version 6.1; DNAstar) was used to analyze electrophorerograms and assemble contigs.

**Table 1:** Sets of external and internal primers used for two-round PCR.

Primer	Nucleotides sequence (5′–3′)	Product size (bp)	Target	Reference
Primers for the first round of amplification				
S-rna- CCHF-F1	acgcccacagtgttctcttgagtg	738	S segment	https://doi.org/10.31082/1728-452X-2018-195-9-54-60
S-rna-CCHF-R1	caaggcctgttgcracaagtgctat			https://doi.org/10.31082/1728-452X-2018-195-9-54-60
M-CCHF-Kuhn-F	caaagaaatacttgcggcacg	956	M segment	https://doi.org/10.1007/s00705-004-0354-3
M-CCHF-NCB-R1	cctyttacaccaytctagyargccttc			https://doi.org/10.31082/1728-452X-2018-195-9-54-60
L-CCHF-NCB-F1	cttamgaggatgctrtctgacaa	821	L segment	https://doi.org/10.31082/1728-452X-2018-195-9-54-60
L-CCHF-NCB-R1	ttgttagarccrtataagaatgttga			https://doi.org/10.31082/1728-452X-2018-195-9-54-60
Primers for the second round of amplification				
Burt- CCHF-F1	tggacaccttcacaaactc	536	S segment	https://doi.org/10.1016/s0166-0934(97)00182-1
Burt- CCHF-R1	gacaaattccctgcacca			
M-CCHF-NCB-F2	tcagtacgtaagtgttaactttgag	847	M segment	https://doi.org/10.31082/1728-452X-2018-195-9-54-60
M-CCHF-NCB-R2	ccttgaggnaangtcaagattat			https://doi.org/10.31082/1728-452X-2018-195-9-54-60
L-CCHF-NCB-F2	tggagayggtgatgtgtttacagc	608	L segment	https://doi.org/10.31082/1728-452X-2018-195-9-54-60
L-CCHF-NCB-R2	gctgcatatgyctttctatycctgt			https://doi.org/10.31082/1728-452X-2018-195-9-54-60

#### Phylogenetic analysis

The nucleotide sequences were aligned using the MUSCLE algorithm (17). Phylogenetic analysis was performed using the MEGA-X software (version 10.2.6) (18), and the evolutionary history was determined by the maximum likelihood (ML) method (19) with 1,000 bootstrap replicates. The identification of clades was carried out as previously described (20, 21). The Africa 4 clade was added to the S gene phylogenetic tree in accordance with the data of M. P. Sánchez-Seco et al. (22).

## Results, discussion

All investigated ticks were collected in the field or removed from agricultural and wild animals over two seasons in the spring and fall of 2021–2022. The spring and fall tick collections were planned depending on weather conditions. We tried to choose days with the most optimal ambient temperature, when the greatest emergence of famished ticks was observed. It is considered the most favorable weather conditions for the emergence of ixodid ticks when the average ambient temperature is 16°C–26°C, especially for ticks of the genus *Dermacentor* (23). As for ticks of the genus *Hyalomma*, they are active throughout warm period of the year, from April to October, reaching maximum numbers in May–June (9). Consequently, the increase in the incidence of CCHF in Kazakhstan is observed precisely in May and June (24).

Out of the 974 ticks that were collected in the western area of Kazakhstan (Aktobe, Atyrau, Mangystau regions): 628 ticks were removed from camels, 90 ticks—from cows, 248 ticks—from large gerbils and their burrows and only 8 ticks in the field. Ticks of the genus *Hyalomma* were dominant in these three investigated regions. The number of collected ticks, species and pools are presented in Table 2.

**Table 2:** Tick species collected in the western regions of Kazakhstan in 2021–2022.

Tick species	Number of ticks in regions (number/pools)			Total number of ticks (number/pools)
	Aktobe region	Atyrau region	Mangystau region	
*Dermacentor marginatus*	7/2	56/11	1/1	64/14
*Hyalomma asiaticum*	159/36	170/43	55/13	384/92
*H. scupense*	247/51	0	0	247/51
*H. excavatum*	0	0	110/22	110/22
*H. dromedarii*	0	0	49/15	49/15
*Haemaphysalis erinacei*	0	94/17	0	94/17
*Ixodes laguri*	0	1/1	0	1/1
*Rhipicephalus schulzei*	0	25/4	0	25/4
Total	413/89	346/76	215/51	974/216

**Figure 1:**
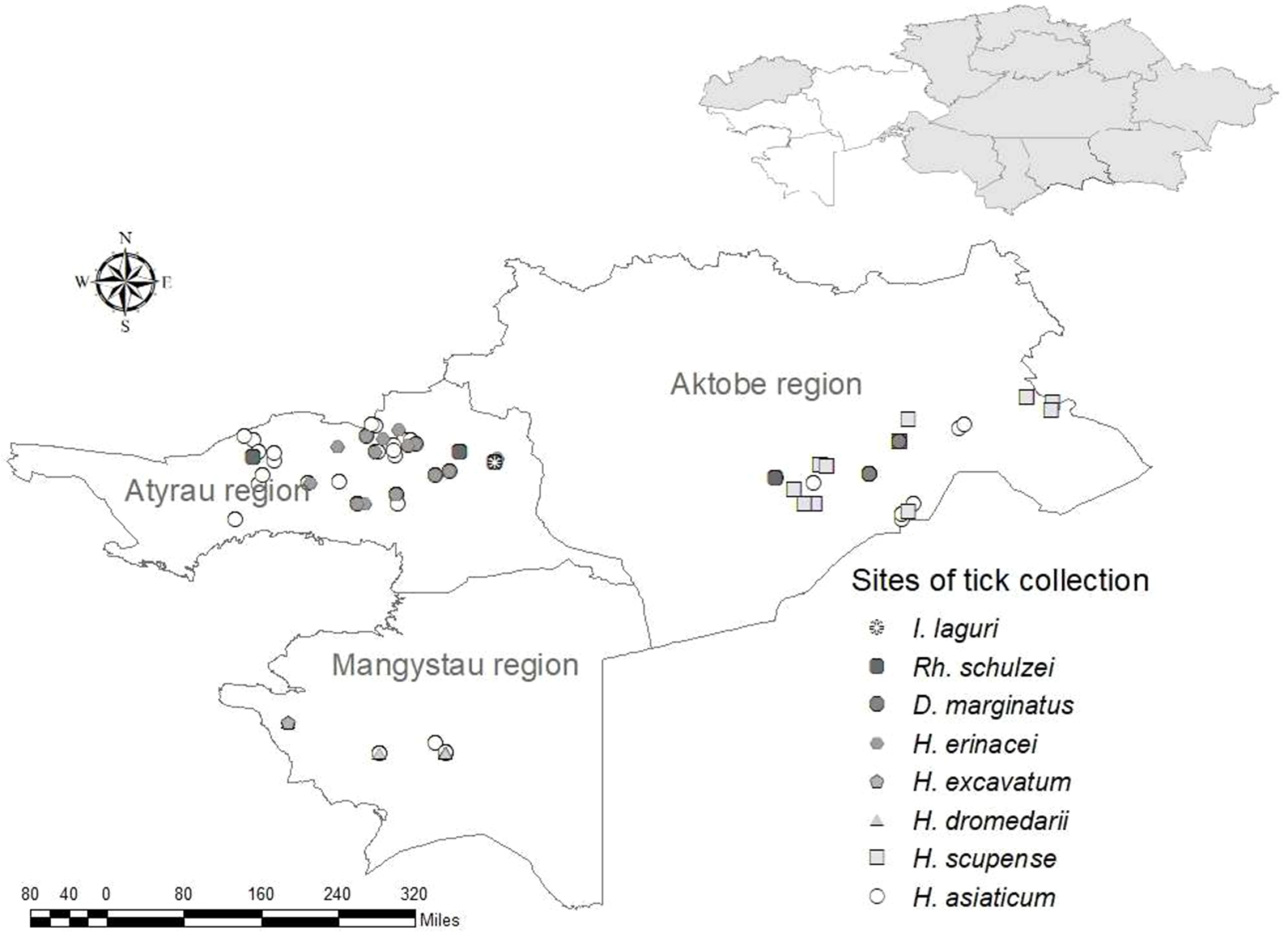
Tick collection sites in the western regions of Kazakhstan.

Ticks of the *Hyalomma* genus are widespread in Kazakhstan and play an important role in the transfer of pathogens of many types of human and animal infections, including the CCHF virus (24, 25). *Hyalomma asiaticum* is the most widespread of the ticks of this genus known for the western area of the countryand was discovered there and collected by us in all three regions. This species is widely spread not only in Kazakhstan, but also in the adjacent territories of Kyrgyzstan, Uzbekistan, as well as in other countries of Central Asia (26–28), where it is a vector of the pathogens of many infectious diseases (9, 29, 30). *H. excavatum* and *H. dromedari* were collected only in the Mangystau region, and *H. scupense—*only in Aktobe region. *Dermacentor marginatus* was also collected by us in all three western regions, but only one specimen was found in the Mangystau region. This find is likely accidental, as this species is not usually live in this region. *Haemaphysalis erinacei*, *Ixodes laguri* and *Rhipicephaluss schulzei* are burrowing ticks that were found and collected from wild animals, mainly from rodents, their number was low and they were collected only in the Atyrau region (Figure 1).

The southern area of Kazakhstan is endemic for CCHF, where human cases of this disease are reported annually, and includes three regions: Kyzylorda, Turkestan and Zhambyl. The continued circulation of the CCHF virus in these regions is facilitated by favorable climatic conditions for vectors and their hosts and intensive breeding of farm animals.

Out of the 3,527 ticks that were investigated from the southern area of the country (Kyzylorda, Turkestan and Zhambyl regions): 566 ticks were removed from agricultural animals, 1,190 ticks—from large gerbils and their burrows, 153 ticks were collected from animal housing facilities and 1,618 ticks—in the field. The diversity of the local natural and climatic landscape provides habitat for a large number of tick species. Ticks of the genus *Hyalomma* were also the most numerous in the southern area of Kazakhstan (Table 3).

**Table 3:** Tick species collected in the southern regions of Kazakhstan in 2021–2022.

Tick species	Number of ticks in regions (number/pools)			Total number of ticks (number/pools)
	Kyzylorda region	Turkestan region	Zhambyl region	
*Dermacentor niveus*	198/40	0	160/26	358/66
*Haemaphysalis erinacei*	67/8	0	0	67/8
*Hyalomma anatolicum*	1,085/105	61/13	29/6	1,175/124
*H. asiaticum*	599/140	433/73	31/7	1,063/220
*H. scupense*	715/129	34/8	0	749/137
*Rhipicephalus anulatus*	4/1	16/2	0	20/3
*Rh. pumilio*	34/10	0	0	34/10
*Rh. turanicus*	4/2	34/7	0	38/9
*Argas persicus*	4/1	8/2	0	12/3
*Ornithodoros tartakovsky*	1/1	0	10/2	11/3
Total	2,711/437	586/105	230/41	3,527/583

**Figure 2:**
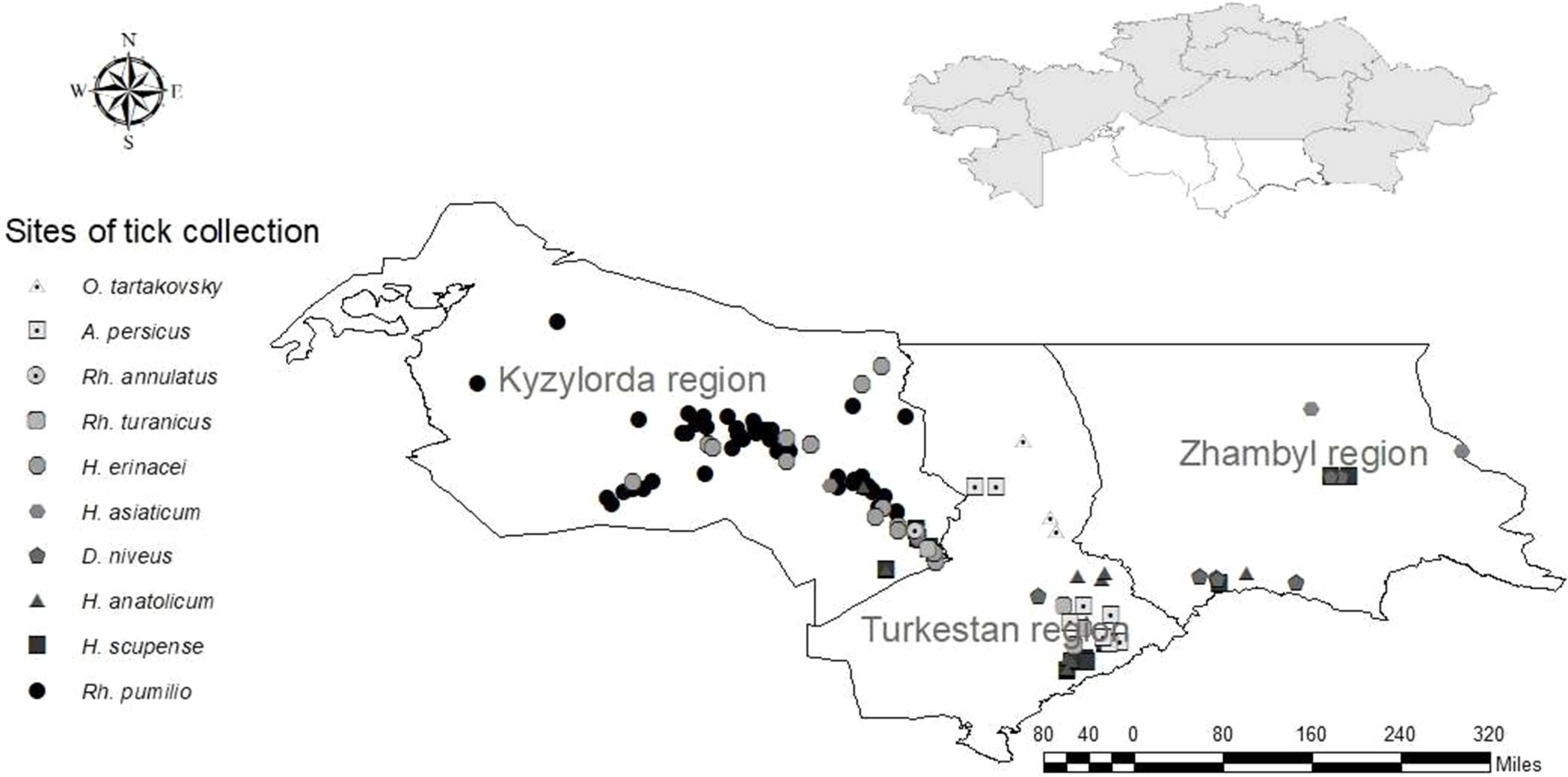
Tick collection sites in the southern regions of Kazakhstan.

*H. asiaticum* and *H. anatolicum* occupy a dominant position in distribution and were also discovered by us in all three southern regions of the country. *H. scupense*, which is also widespread in the south of Kazakhstan, was collected in the Kyzylorda and Turkestan regions and was inferior in number to the two previous species. *Dermacentor niveus* was collected in the Kyzylorda and Zhambyl regions, and ticks of the genus *Rhipicephalus* were collected in the Turkestan and Kyzylorda regions (Figure 2). In addition to ixodid ticks, we collected and investigated for the presence of the CCHF virus two species of argasid ticks—*Argas persicus*, collected from poultry keeping facilities, and *Ornithodoros tartakovskiy—*from rodent's burrows.

**Figure 3:**
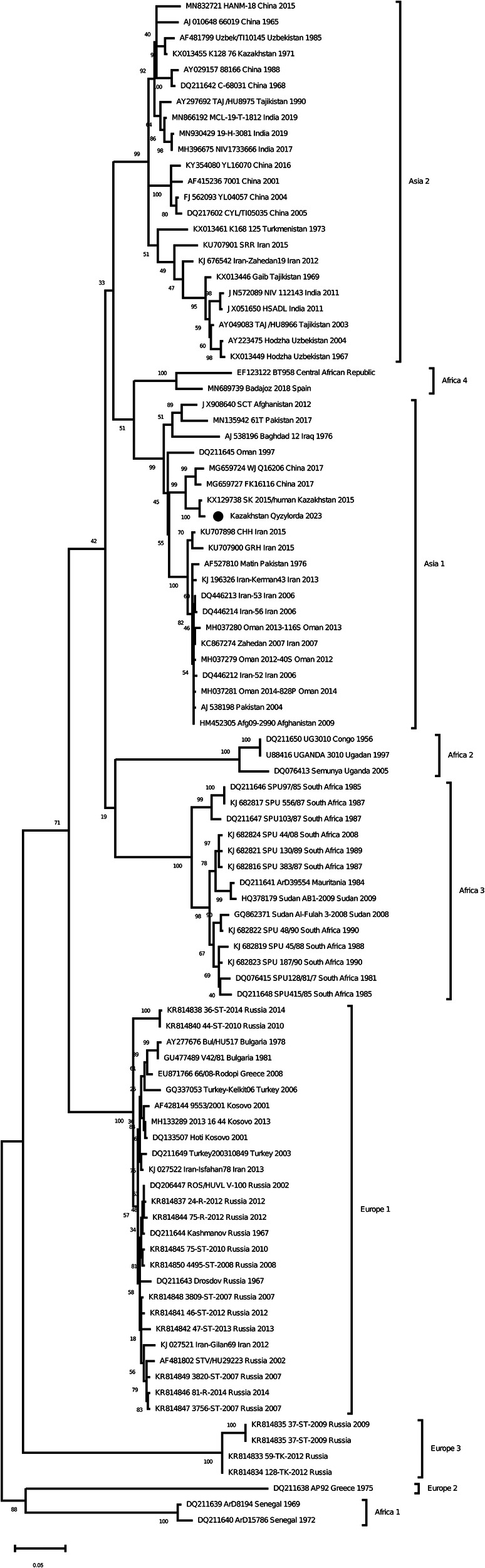
Phylogenetic analysis of the CCHF virus (32, 33). The phylogenetic tree was constructed using oligonucleotide sequences of the S gene of the CCHF virus from the ticks of *Hyalomma scupense*, collected in the southern regions of Kazakhstan, and shown as a black dot. Oligonucleotide sequences were aligned using the software MEGA version 6.0.

**Figure 4:**
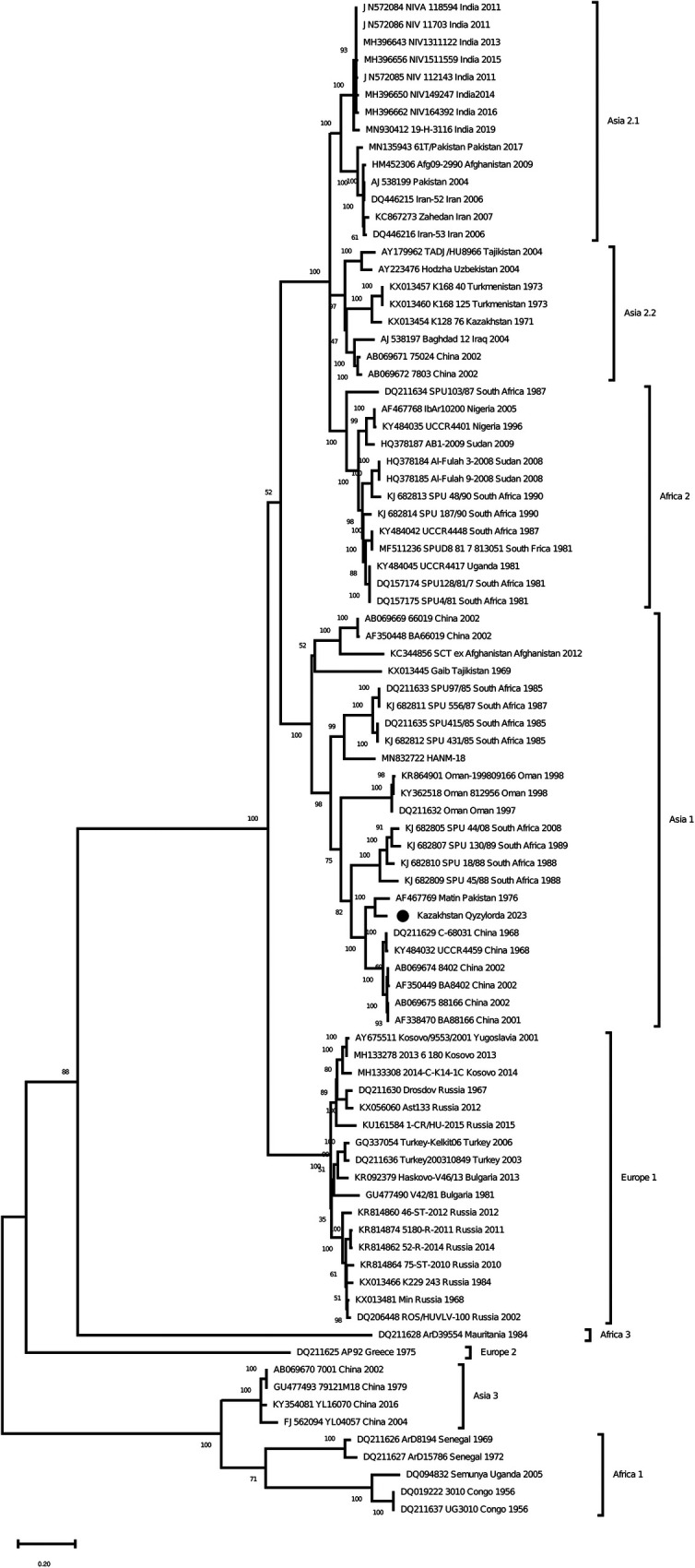
Phylogenetic analysis of the CCHF virus. The phylogenetic tree was constructed using oligonucleotide sequences of the M gene of the CCHF virus from the ticks of *Hyalomma scupense*, collected in the southern regions of Kazakhstan, and shown as a black dot. Oligonucleotide sequences were aligned using the software MEGA version 6.0.

**Figure 5:**
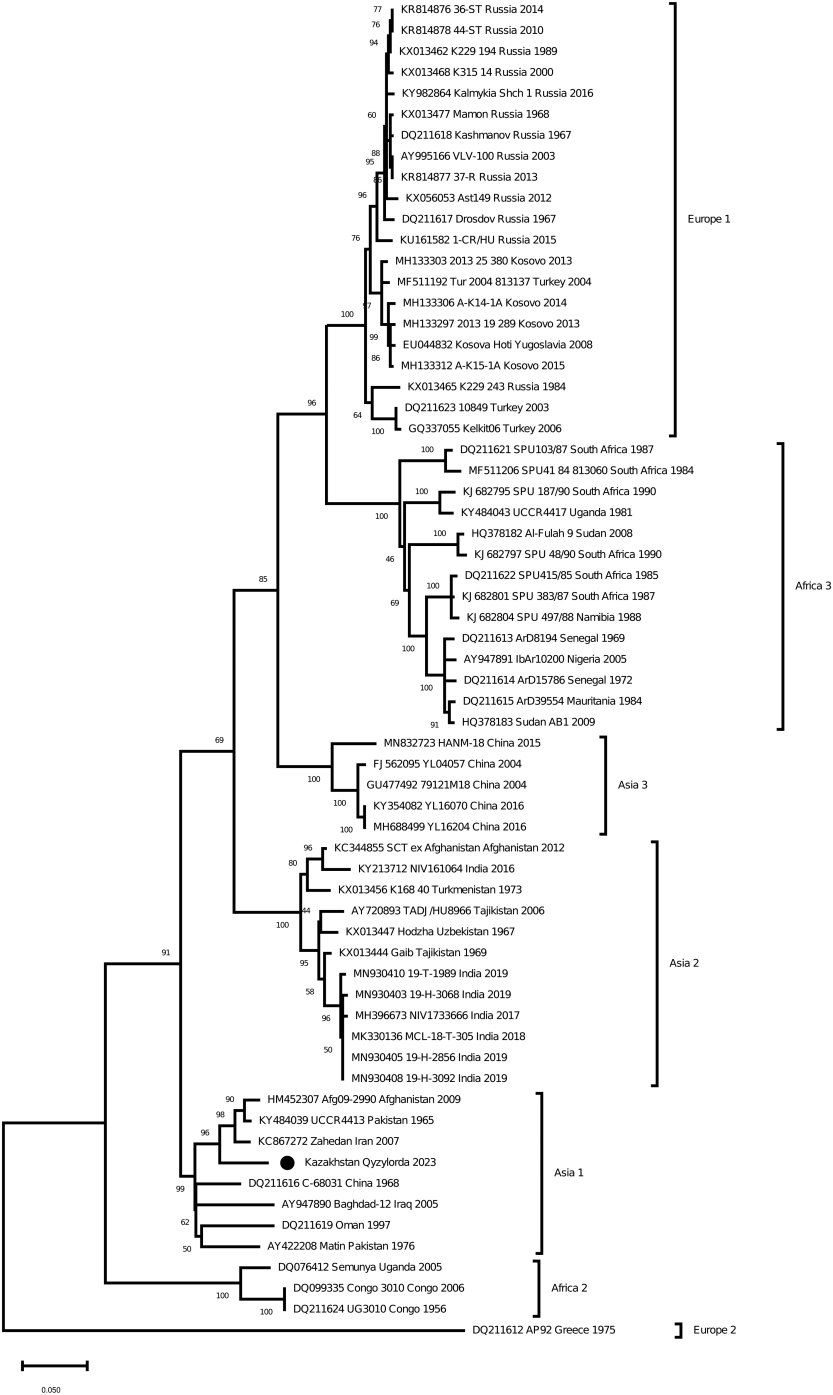
Phylogenetic analysis of the CCHF virus. The phylogenetic tree was constructed using oligonucleotide sequences of the L gene of the CCHF virus from the ticks of *Hyalomma scupense*, collected in the southern regions of Kazakhstan, and shown as a black dot. Oligonucleotide sequences were aligned using the software MEGA version 6.0.

As a result of Real-time qPCR, 1 positive pool for the CCHF virus was obtained out of 799 investigated pools of ticks. The positive pool was from the ticks of *Hyalomma scupense*, which were collected in the endemic for CCHF Kyzylorda region. It was early reported that ticks of this species can transmit the CCHF virus in this region (31–33). The partial sequences of the S, M and L segments were obtained for this sample (GenBank accession numbers OR633376, OR633377 and OR633378). Phylogenetic analysisclustered the analyzed samples into the Asia 1 clade (Figures 3–5). The S-segment sequences (GenBank accession number OR633376) showed 99.43% identity at the nucleotide and aminoacid level, respectively, to the strain (GenBank accession number KX129738) previously isolated froma patient with CCHF in the Turkestan region (34) (Figure 3).

The results confirm the circulation of the CCHF virus in the investigated endemic areas (34). The detection of the virus in the ticks of *Hyalomma scupense* has important epidemiological significance, since this species is a pasture-stall ectoparasite with a one- or two-host development cycle. Ticks of this species spend their life cycle in animal housing facilities and in the vicinity of populated areas. Their larvae and nymphs can feed on blood and molt on the same animal, as a result of which the emerging imagos, having had enough, are moved to the ground for reproduction in populated areas or its environs, where an exchange of ectoparasites between animals occurs. The indicated features of the life cycle of this species of ticks must be taken into account when carrying out anti-tick treatments.

The presence of geographically distant but genetically similar strains suggests that the CCHF viruses are spread either through the trade in livestock or through migratory birds. This hypothesis requires further in-depth investigation to confirm or disprove it.

The discovery of Asia 1 (clade VI) serogroup of CCHF virus in ticks in the Kyzylorda region, which has genetic similarity to strains isolated from a patient in 2015 in the Turkestan region, is important in understanding the evolution of this virus in the study areas.

## Data Availability

The datasets presented in this study can be found in online repositories. The names of the repository/repositories and accession number(s) can be found in the article/Supplementary Material.
